# Validation of non-invasive cerebrovascular pressure reactivity and pulse amplitude reactivity indices in traumatic brain injury

**DOI:** 10.1007/s00701-019-04169-9

**Published:** 2019-12-18

**Authors:** Leanne A. Calviello, András Czigler, Frederick A. Zeiler, Peter Smielewski, Marek Czosnyka

**Affiliations:** 1 Division of Neurosurgery, Department of Clinical Neurosciences, Addenbrooke’s Hospital, University of Cambridge, Cambridge, U.K.; 2 Division of Neurosurgery, Department of Clinical Neurosciences, Cambridge Biomedical Campus, University of Cambridge, Cambridge, U.K.; 3 Department of Neurosurgery, University of Pecs, Pécs, Hungary; 4 Division of Anesthetics, Addenbrooke’s Hospital, University of Cambridge, Cambridge, U.K.; 5 Section of Neurosurgery, Department of Surgery, Rady Faculty of Health Sciences, University of Manitoba, Winnipeg, Canada

**Keywords:** Cerebral autoregulation, Traumatic brain injury, Pressure reactivity index, Intracranial pressure, Transcranial Doppler, Cerebral arterial blood volume

## Abstract

**Background:**

Two transcranial Doppler (TCD) estimators of cerebral arterial blood volume (CaBV) coexist: continuous outflow of arterial blood outside the cranium through a low-pulsatile venous system (continuous flow forward, CFF) and pulsatile outflow through regulating arterioles (pulsatile flow forward, PFF). We calculated non-invasive equivalents of the pressure reactivity index (PRx) and the pulse amplitude index PAx with slow waves of mean CaBV and its pulse amplitude.

**Methods:**

About 273 individual TBI patients were retrospectively reviewed. PRx is the correlation coefficient between 30 samples of 10-second averages of ICP and mean ABP. PAx is the correlation coefficient between 30 samples of 10-second averages of the amplitude of ICP (AMP, derived from Fourier analysis of the raw full waveform ICP tracing) and mean ABP. nPRx is calculated with CaBV instead of ICP and nPAx with the pulse amplitude of CaBV instead of AMP (calculated using both the CFF and PFF models). All reactivity indices were additionally compared with Glasgow Outcome Score (GOS) to verify potential outcome-predictive strength.

**Results:**

When correlated, slow waves of ICP demonstrated good coherence between slow waves in CaBV (>0.75); slow waves of AMP showed good coherence with slow waves of the pulse amplitude of CaBV (>0.67) in both the CFF and PFF models. nPRx was moderately correlated with PRx (*R* = 0.42 for CFF and *R* = 0.38 for PFF; *p* < 0.0001). nPAx correlated with PAx with slightly better strength (*R* = 0.56 for CFF and *R* = 0.41 for PFF; *p* < 0.0001). nPAx_CFF showed the strongest association with outcomes.

**Conclusions:**

Non-invasive estimators (nPRx and nPAx) are associated with their invasive counterparts and can provide meaningful associations with outcome after TBI. The CFF model is slightly superior to the PFF model.

## Introduction

The pressure reactivity index (PRx) is a common descriptor of cerebrovascular reactivity (CVR) following traumatic brain injury (TBI). PRx has become essential to patient prognostication, with negative or zero values of PRx indicative of favorable outcome and positive values indicative of poor outcome [1]. Traditionally relying on the input from invasive, continuous arterial blood pressure (ABP) and intracranial pressure (ICP) monitors, PRx is considered to be an invasively quantified surrogate marker of cerebral autoregulation (CA) that accounts for changes in intracerebral blood volume attributable to either vasodilation or vasoconstriction [2].

The pulse amplitude index (PAx) is another index of cerebrovascular reactivity, which theoretically can outperform PRx when the compliance of the cranial space is increased (i.e., after craniectomy, with CSF leakages, etc.). It correlates the changes in the pulse amplitude of ICP (AMP) with changes in mean ABP (as the moving correlation coefficient of 30 samples of 10-second averages of AMP and mean ABP). Both PRx and PAx can be only calculated when ICP is monitored. Since ICP monitoring usually occurs over a few days or even weeks after TBI, PRx and PAx may be used for long-term management of patients (i.e., optimalcerebral perfusion pressure (CPP) -oriented therapy [3–6]).

Indices of cerebral autoregulation can be calculated directly with TCD monitoring. The mean flow index (Mx) or the systolic flow index (Sx) show stronger performance than PRx (the moving correlation coefficients of 30 samples of 10-second averages of mean or systolic cerebral blood flow velocity (FV) and mean cerebral perfusion pressure (CPP)) [7, 8]. However, TCD monitoring is intermittent, (30 minutes to a few hours daily), whereas ICP monitoring is continuous. This is associated with the difficulty to maintain the continuous insonation of cerebral vessels that is essential to the calculation of TCD indices; Sx and Mx are probably more accurate than PRx and PAx, but the latter indices can be used continuously.

Changes in cerebral arterial blood volume can be calculated in two ways. Equation 1 presumes that pulsatile inflow through the basal arteries is equilibrated by non-pulsatile blood outflow through the dural sinuses, creating the continuous flow forward model (CFF). Equation 2 presumes that the inflow of arterial blood is equilibrated by pulsatile flow forward through the regulating arterioles (the pulsatile flow forward model, PFF) [9].

1 $$ \Delta {C}_a{BV}_{CFF}(t)=\underset{t_0}{\overset{t}{\int }}\left( CB{F}_a(s)-{meanCBF}_a\right) ds $$

2 $$ \varDelta {C}_a{BV}_{PFF}(t)={\int}_{t_0}^t\left( CB{F}_a(s)-\frac{ABP(s)}{CVR}\right) ds $$

where *s* is the arbitrary time variable of integration*, CBF*_*a*_ is cerebral blood flow, *ABP* is arterial blood pressure, and *CVR* is cerebrovascular resistance [9].

There is great clinical interest in the application of non-invasive metrics (particularly more accurate surrogate measures of PRx and PAx) during the subacute and long-term phases of TBI care, where invasive ICP monitoring is no longer present and is thus unable to influence patient management or contribute to traditional PRx and/or PAx evaluation. Although the established Mx and Sx are TCD metrics of cerebrovascular reactivity, they are in composition not true direct surrogates of PRx and PAx, even if there is a moderate correlation between them. The purpose of nPRx and nPAx is to provide, as closely as possible, non-invasive measures for PRx and PAx by modeling the constituent components of invasively-derived PRx and PAx using noninvasive TCD-based models of pulsatile CaBV as a direct surrogate for ICP. Doing so provides nPRx and nPAx indices which are more similar in method of derivation and physiologic composition than other TCD metrics (i.e., Mx and Sx).

This retrospective study seeks to explore the utility of nPRx and nPAx (calculated with both the CFF and the PFF models of CaBV) by correlating them with the established cerebrovascular reactivity markers PRx and PAx. As slow waves between ICP and CaBV are well-synchronized (due to ICP pulsatility and CaBV modifications being triggered simultaneously during the cardiac cycle), it was presumed that the CFF and PFF models could evaluate cerebrovascular reactivity in the absence of invasive ICP monitoring. A secondary aim of this work is to correlate all of the aforementioned indices with patient outcome according to the Glasgow Outcome Score (GOS).

## Materials and methods

### Patients

About 273 severely head-injured patients (218 males and 55 females with an average age of 33 years [range: 3–77 years]) were admitted to the Neurosciences Critical Care Unit (NCCU) at Addenbrooke’s Hospital, Cambridge, U.K., between 1992 and 2012. All patients were managed in accordance with an ICP/CPP-oriented protocol designed to maintain ICP below 20 mm Hg. The exact protocol changed several times over the monitoring period, but its essential components were stable [10].

### Monitoring

All patients underwent both invasive (ABP and ICP) and daily non-invasive monitoring (TCD) while admitted to NCCU. Raw data signals from select monitoring devices were recorded and electronically stored using WREC software (Warsaw University of Technology) and ICM+ software (Cambridge Enterprise, Cambridge, U.K.; http://www.neurosurg.cam.ac.uk/icmplus).

ABP was continuously monitored both invasively [from the radial artery using a pressure monitoring kit (Baxter Healthcare C.A., U.S.A.; Sidcup, U.K.)] and noninvasively. ICP was monitored using an intraparenchymal probe with strain gauge sensors (Codman & Shurtleff, M.A., U.S.A. or Camino Laboratories, C.A., U.S.A.). Blood flow velocities were monitored from the middle cerebral artery (MCA) with a 2 MHz probe (Multi Dop X4, DWL Elektronische Systeme, Sipplingen, Germany). Raw TCD data recordings were sampled within the entire patient cohort (295 individual recordings) with an average continuous monitoring duration of 35 minutes. Of the patients receiving multiple TCD monitoring sessions, all recordings were utilized where signal quality was adequate. TCD measurements were intermittently performed anywhere between the first 24 hours of admission and before final removal of intraparenchymal ICP sensors. The exact period and availability of TCD monitoring varied on an individual basis.

This study was conducted as a retrospective analysis of a prospectively-maintained database cohort, in which high-frequency clinical neuromonitoring data had been archived. The monitoring of brain modalities was conducted as a part of standard Neurosciences Critical Care Unit (NCCU) patient care using an anonymized database of physiological monitoring variables in neurocritical care. Data on age, injury severity, and clinical status at hospital discharge were recorded at the time of monitoring on this database, and no attempt was made to re-access clinical records for additional information (REC 97/291). Since all data was extracted from the hospital records and fully anonymized, no data on patient identifiers were available, and need for formal patient or proxy consent was waived. Within our institution, patient data may be collected with waiver of formal consent, as long as it remains fully anonymized, with no method of tracing this back to an individual patient. Patient physiologic, demographic, and outcome data were collected by the clinicians involved with patient care and subsequently recorded in an anonymous format. This anonymous data is then provided for future research purposes. Such data curation remains within compliance for research integrity as outlined in the U.K. Department of Health - Governance Arrangements for Research Ethics Committees (GAfREC), guidelines, section 6.0 [11].

### Data processing

Processing of raw data signals utilized ICM+ software (Cambridge Enterprise, Cambridge, U.K.; http://www.neurosurg.cam.ac.uk/icmplus). Signal artifact removal was first conducted with signal cropping tools within ICM+. CPP was determined from the difference between raw ABP and ICP signals.

Primary analysis involved the calculation of time-averaged mean values for ABP, ICP, cerebral blood flow velocity (FV), CPP, CaBV_CFF (according to Eq. 1, taking the FV signal instead of CBF), and CaBV_PFF (according to Eq. 2, taking the FV signal instead of CBF). Substituting CBF in Eqs. 1 and 2 with blood flow velocities has a consequence; estimators of blood volume are presented as blood volume per 1 cm^2^ of cross-sectional area of the vessel. Also, the arbitrary choice of t_0_ within the calculation window of each interval containing 10 to 20 heartbeats produces the effect that only the relative changes of cerebral arterial blood volume can be observed with the CaBV(t) signals. The amplitudes of the fundamental frequencies of CaBV_CFF and CaBV_PFF pulse waveforms (i.e., for a frequency equivalent to heart rate) were also calculated as AMP_CFF and AMP_PFF, respectively.

Mean values of the listed parameters were calculated during 10-second time windows and were updated every 10 seconds to emphasize vasogenic slow wave fluctuations and to eliminate overlap. A coherence module was calculated between series of 10-second averages of ICP and CaBV_CFF and CaBV_PFF in the frequency band ranging from 0.005 to 0.05 Hz. The same calculations were applied to time series of AMP, AMP_CFF, and AMP_PFF.

Final data processing involved the calculations from the primary analysis, with the addition of PRx (the correlation between ABP and ICP), nPRx_CFF (the correlation between CaBV_CFF and ABP), and nPRx_PFF (the correlation between CaBV_PFF and ABP). Non-invasive PAx was calculated by correlating ABP with either AMP_CFF or AMP_PFF (nPAx_CFF and nPAx_PFF, respectively). Each of these parameters was calculated utilizing a 300-second time window, updated every 10 seconds.

Post-processing, all 10-second by 10-second data were exported from each patient to separate comma-separated variable (CSV) files for further statistical analysis.

### Statistics

All statistical analyses were conducted utilizing R software (R Core Team [2017]; R: a language and environment for statistical computing. R Foundation for Statistical Computing, Vienna, Austria, URL https://www.R-project.org/). Grand means of and descriptive statistics for each parameter were calculated. Data were normally distributed. Descriptive analyses were applied to the coherences between ICP slow waves and CaBV and between the AMP and AMP_CaBV series.

Linear regression techniques were employed to describe the following relationships in the entire cohort: PRx vs. nPRx_CFF, PRx vs. nPRx_PFF, PAx vs. nPAx_CFF, and PAx vs. nPAx_PFF. Goodness of fit was reported utilizing the Pearson correlation coefficient (*R*). Agreement between the parameters was assessed with the Bland-Altman method.

Each of the above indices was also correlated with dichotomized Glasgow Outcome Score (GOS) data (favorable vs. unfavorable outcome). Favorable outcome was classified by GOS of 4 (moderate disability) and 5 (mild to no disability). Unfavorable outcome was classified by GOS of 1 (dead), 2 (vegetative state), or 3 (severe disability). The strength of the relationship between each index and outcome was reported via area under the receiver operating curve (AUC), with bold AUCs reaching *p* <0.05 (statistical significance identified by the DeLong test). *p* values between groups were compared with *t*- and Mann-U tests.

## Results

**Fig. 1:**
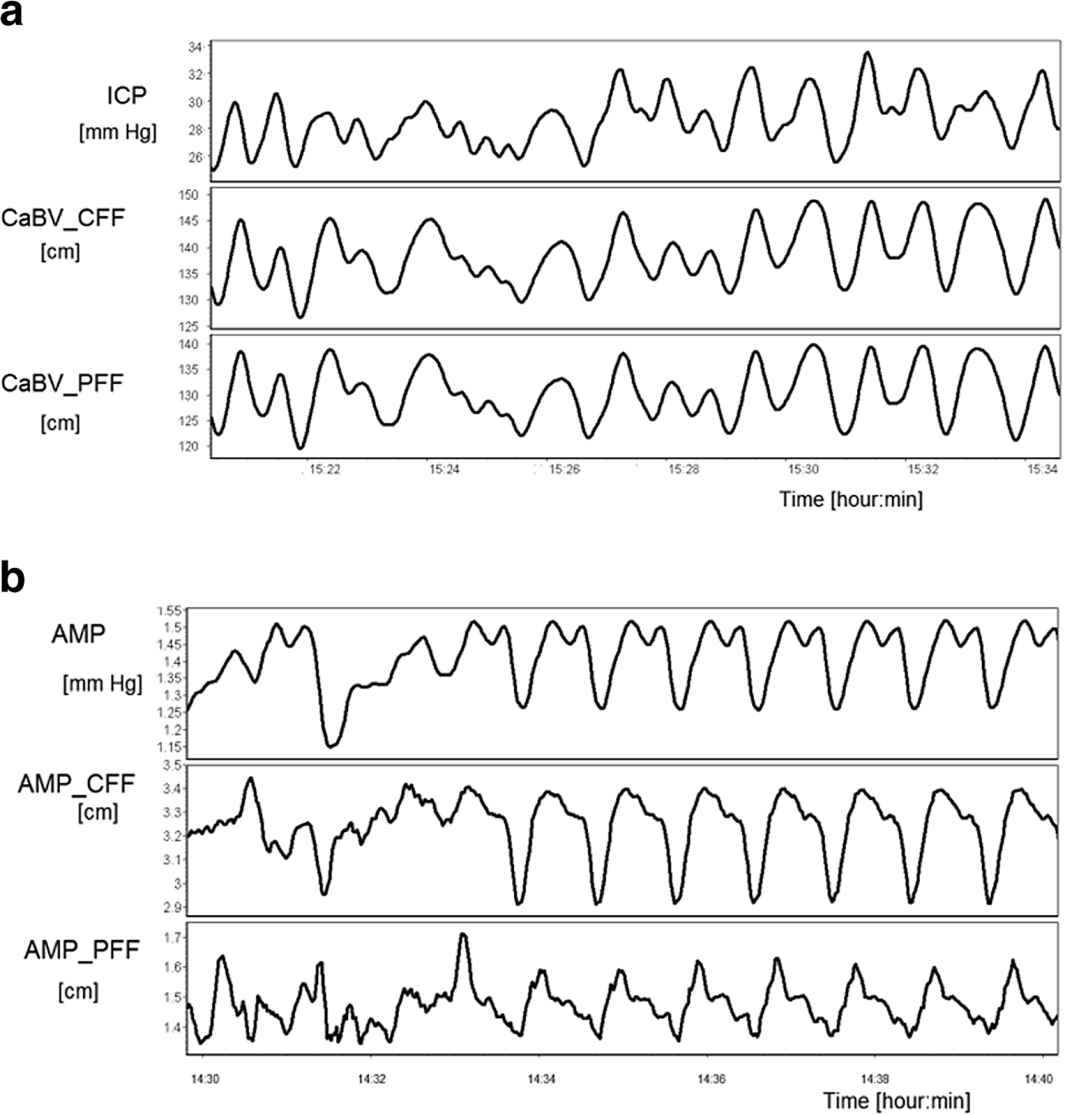
Examples of good synchronization of mean ICP and CaBV time series in the frequency range of slow waves (Fig. 1A). Figure 1B demonstrates good synchronization of the AMP time series with AMP_CFF and AMP_PFF

Table 1 summarizes descriptive statistics for the entire cohort of TBI patients. Slow waves of ICP and CaBV in most cases appeared well-synchronized in time (Fig. 1A, top panel). The same observation can be made for time series of AMP, AMP_CFF, and AMP_PFF (Fig. 1B, bottom panel). The average of the module of coherence functions in low frequency limits (0.005 to 0.05 Hz) is presented in Table 2.

**Table 1:** Mean cerebral hemodynamic parameters in TBI

Parameter	Mean	Range	Standard deviation
Age [years]	33.14	3.0–77.0	± 15.96
Favorable: unfavorable outcome	132:122	1–5	–
Admission GCS (median)	6	1–15	IQR 4
ABP [mm Hg]	91.36	58.61–147.57	± 12.08
ICP [mm Hg]	18.12	− 3.27-75.69	± 9.92
CPP [mm Hg]	73.61	20.63–109.55	± 13.20
FV [cm/s]	63.41	19.67–168.79	± 25.64
PRx	0.02	− 0.65–0.96	± 0.27
PAx	− 0.10	− 0.93–0.76	± 0.20
nPRx_CFF	0.16	− 0.41–0.80	± 0.20
nPRx_PFF	− 0.21	− 0.69–0.45	± 0.19
nPAx_CFF	− 0.07	− 0.45–0.56	± 0.14
nPAx_PFF	− 0.06	− 0.44–0.62	± 0.13

*ABP* arterial blood pressure, *CaBV_CFF* cerebral arterial blood volume calculated with the continuous flow forward method, *CaBV_PFF* cerebral arterial blood volume calculated with the pulsatile flow forward method, *cm/s* centimeters per second, *CPP* cerebral perfusion pressure, Favorable: Unfavorable Outcome – Glasgow Outcome Score [Favorable: GOS 4–5 (moderate-mild, or no disability); Unfavorable: GOS 1–3 (dead, vegetative state, or severe disability)], *FV* cerebral blood flow velocity, *Admission GCS* - Glasgow Coma Score on admission, *mm Hg* millimeters of mercury, *IQR* interquartile range, *nPRx_CFF* non-invasive PRx calculated with the continuous flow forward method, *nPRx_PFF* non-invasive PRx calculated with the pulsatile flow forward method, *PAx* pulse amplitude index, and *PRx* pressure reactivity index.

**Table 2:** Coherences between variables within the frequency range of 0.005–0.05 Hz

Variables	Module of coherence < 0.005–0.05 Hz >	95% Confidence intervals
ICP vs. CaBV_CFF	0.765	0.748–0.782
ICP vs. CaBV_PFF	0.758	0.741–0.776
AMP vs. AMP_CFF	0.73	0.718–0.747
AMP vs. AMP_PFF	0.678	0.665–0.692

**Fig. 2:**
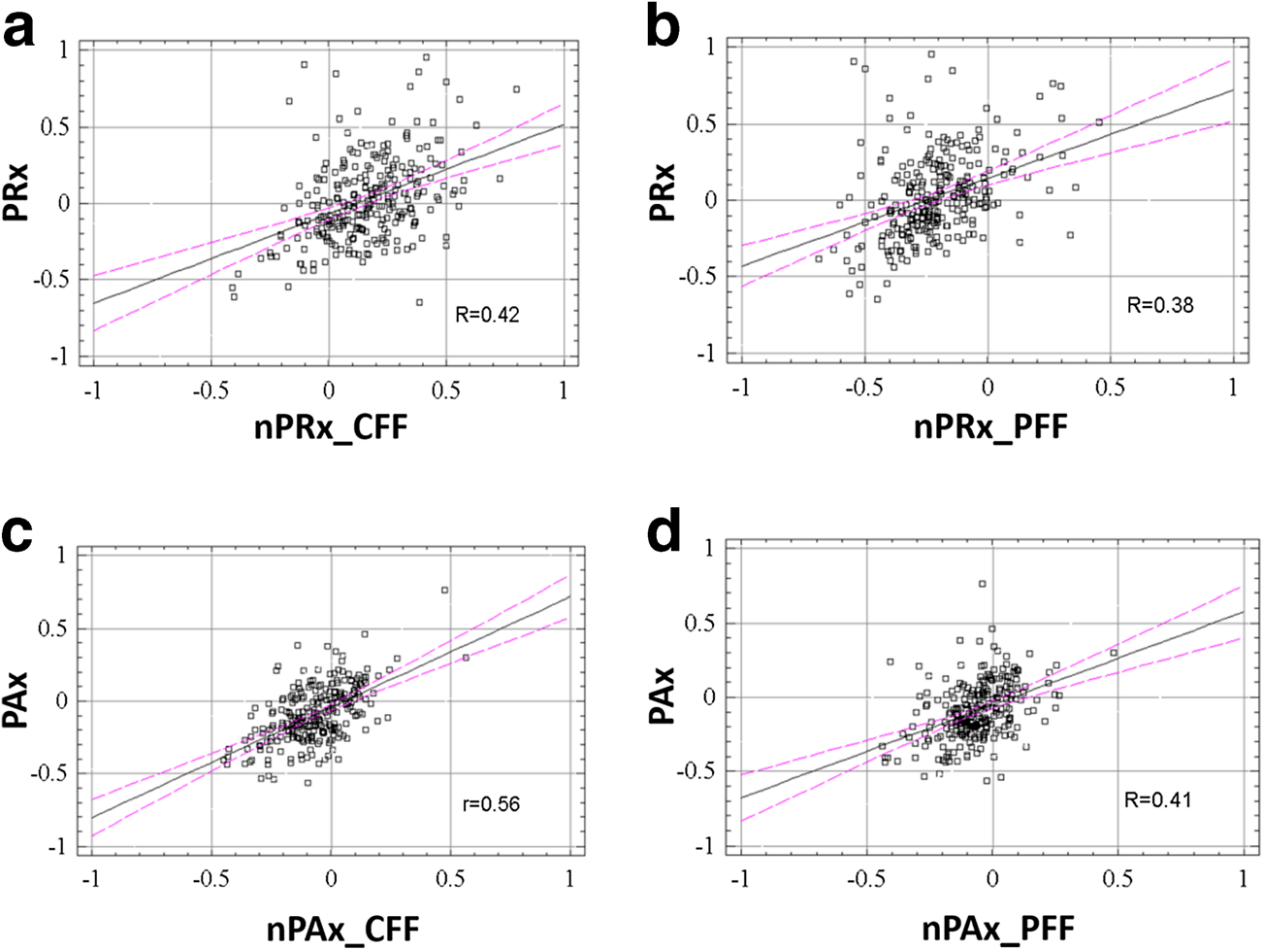
Scatterplots of PRx vs. nPRx and PAx vs. nPAx calculated by the different CaBV models. Pearson correlation coefficients are given. The correlation between PAx and nPAx_CFF is significantly the strongest (*p* <0.003). Correlations between traditional parameters and derived parameters based on CFF models are stronger than those based on PFF models (*p* <0.01 for nPAx and *p* <0.076 for nPRx)

The correlations between PRx and nPRx and those between PAx and nPAx are only moderately strong (although the *R* value is significantly non-zero at *p* <0.0001). Scatterplots and correlation coefficients for each model (calculated with either the CFF or PFF methods) are shown in Fig. 2. Table 3 includes the results of Bland-Altman analysis for invasive and non-invasive reactivity indices. The majority of data points were clustered around the mean for each of the modeled pairs with few outliers. It must be noted that the outliers derived from the results shown in Fig. 2 are due to the varying blood pressures exhibited by patients in the TBI database.

**Table 3:** Bland-Altman agreement between PRx-nPRx and PAx-nPAx

	Critical difference	Lower limit	Mean difference	Upper limit
PRx vs. nPRx_CFF	0.51	− 0.65	− 0.13	0.38
PRx vs. nPRx_PFF	0.52	− 0.28	0.24	0.76
PAx vs. nPAx_CFF	0.33	− 0.36	− 0.03	0.30
PAx vs. nPAx_PFF	0.40	− 0.44	− 0.03	0.37

Finally, all reactivity indices (invasive and non-invasive) were compared in two outcome groups: favorable outcome (*n* = 132) and unfavorable outcome (*n* = 122). About 19 patients were not available for follow-up. The strongest separation was detected for the nPAx_CFF index (Table 4). nPAx_CFF performed the best when compared to outcome but was not significantly different from the other indices when evaluated with the DeLong test.

**Table 4:** Differences between reactivity indices in patients with favorable and unfavorable outcomes at 6 months after TBI

Pressure Reactivity Index	FAVORABLE	UNFAVORABLE	*p* value	*p* value	*AUC*
	Mean and 95% CI	Mean and 95% CI	t-test	Mann-U	*95% CI*
PRx	−0.014 [−0.046;0.018]	0.189 [0.165;0.21]	**0.022**	0.078	**0.564 [0.493–0.635]**
nPRx_CFF	0.137 [0.113;0.161]	0.189 [0.165;0.21]	**0.037**	**0.021**	**0.584 [0.514–0.654]**
nPRx_PFF	−0.242 [−0.26;-0.22]	−0.018 [−0.21;-0.16]	**0.013**	**0.015**	**0.601 [0.531–0.671]**
PAx	−0.134 [−0.159;-0.11]	−0.055 [−0.08;-0.028]	**0.018**	**0.002**	**0.615 [0.546–0.684]**
nPAx_CFF	−0.10 [−0.12;-0.085]	−0.037 [−0.055;-0.019]	**0.0003**	**0.0003**	**0.632 [0.564–0.701]**
nPAx_PFF	−0.076 [−0.09;-0.059]	−0.052 [−0.069;-0.035]	0.164	0.093	0.561 [0.490–0.632]

The strength of the relationship between each index and outcome was additionally reported via area under the receiver operating curve (AUC), with bold AUCs reaching *p* < 0.05.

## Discussion

In our evaluated population of TBI patients, nPRx and nPAx calculated with the CFF model were found to better associate with PRx and PAx. It is likely that the PFF model in general is more susceptible to variations in its key components (i.e., unstable ABP in patients would affect the numerators in Eq. 2) that impact its stability as a calculation method; this effect is consistent with the findings of Eide et al. [12], which discovered weak correlations between ABP and ICP pulse pressure amplitudes and autoregulation indices such as PRx. On the basis of their results [12], as the PFF model is comprised of input from the ABP signal, it is fitting that the nature of the nPRx_PFF index is incompatible with PRx and PAx which are all “noisy” surrogate markers of cerebral autoregulation to begin with. The CFF and PFF models partially account for total cerebral blood volume change, as they are calculated as the difference between systolic and mean cerebral blood flow integrated over a given period of time. Cerebral blood flow velocity as assessed by TCD is a surrogate measure of cerebral blood flow, as the TCD monitoring technique does not directly quantify cerebral circulation. Thus, current applications of these models can only approximate cerebral blood volume change.

PRx ultimately responds to alterations in cerebral blood volume and responds to both ICP and ABP fluctuations as vessel diameter changes. There is the additional possibility that PRx may be inaccurately represented in TBI patients with either low or high levels of ICP, as the index does not describe cerebrospinal fluid compliance, which influences the direction of cerebral blood volume change, and thus measured ICP^3^. As the nPRx and nPAx indices do not rely on information from invasive ICP sensors but rather ABP, they only moderately correlated with traditional PRx and PAx, which are both more commonly associated with ICP. It is important to note that the determination of nPRx and nPAx with the current TCD-based CFF and PFF models cannot definitively describe the relationships between the “true” inputs from cerebral blood flow and either the ABP or the ICP signals.

Our no-ninvasive TCD models of PRx and PAx based on CaBV estimates provide information closer to invasively derived ICP. Further refinement of the nPRx_CFF model in particular will enhance the ability to non-invasively approximate traditional PRx, which has been experimentally validated as a measure of the lower limit of autoregulation [13]. nPRx can be employed for long-term follow-up using continuous, non-invasive ABP (via finger-cuff). Cerebrovascular reactivity during the subacute phase of care can be correlated with long-term autoregulatory status, inclusive of clinical phenotype and chronic neuroimaging changes (i.e., magnetic resonance imaging (MRI)-based cortical atrophy or diffusion tensor imaging (DTI) white matter tract volume). nPRx can inform clinicians of patient autoregulatory status in the absence of neurosurgical placement of invasive monitors; it can be directly calculated from emergency rooms or in remote hospitals without neurosurgical services. Non-invasive determinations of optimal cerebral perfusion pressure (nCPP_opt_) can also benefit from nPRx, as PRx is a key component in the visualization of CPP targets [6].

When comparing both traditional and derived autoregulation indices with outcome, nPAx_CFF trended toward higher AUCs in association with dichotomized 6-month outcomes. We must acknowledge that TCD-based indices such as Mx and Sx and both sets of nPRx and nPAx estimators can only be calculated if patients receive TCD monitoring, which is intermittently applied at best; at present, it is difficult to provide continuous measures of PRx or PAx in the absence of invasive monitoring. Traditional TCD devices such as the DWL Multi Dop X4 require careful placement of TCD probes that are both fragile and very easily disturbed by small movements. Although emerging TCD technology with robotic-assisted probes allows for longer, uninterrupted TCD monitoring, these newer devices are ultimately less popular and too expensive for the majority of centers to obtain for purely research purposes.

### Limitations

The strength of our study is fundamentally limited by our reliance on intermittent TCD recordings that were relatively short in duration and susceptible to motion artifacts. Additionally, statistical analyses were based on grand mean data, which reduces the natural variability within the dataset and can potentially create artificial effects. The correlation coefficient values are in a weak to moderate range for strength. As such, the definitiveness of conclusions from this current study is limited and should not be extrapolated to other TBI populations at this time. Much further multicenter investigation into nPRx and nPAx is required prior to implementation as a clinical bedside measure for cerebrovascular reactivity. It is also worth noting that Mx and Sx have been previously validated as having stronger outcome-predictive power than PRx [12], which we did not address in this study. The rationale for nPRx and nPAx is somewhat artificial at the moment; these indices may become more clinically relevant when the next generation of continuous TCD monitoring devices becomes available. There are differences between TCD-based autoregulation and pressure reactivity [14]; with better technology, it may be useful to explore them jointly.

## Conclusions

With TCD, it is possible to derive non-invasive estimators of PRx and PAx based on cerebral blood volume modeling. Direct clinical application of these non-invasive cerebrovascular reactivity indices is limited by the current state of continuous TCD monitoring technologies, but following further improvements on the auto-focusing of TCD probes and waveform visualization, they may become clinically useful.
